# Surrogate fostering of mice prevents prenatal estradiol-induced insulin resistance *via* modulation of the microbiota-gut-brain axis

**DOI:** 10.3389/fmicb.2022.1050352

**Published:** 2023-01-09

**Authors:** Huihui Wang, Chengliang Zhou, Shuping Gu, Yun Sun

**Affiliations:** ^1^Center for Reproductive Medicine, Renji Hospital, School of Medicine, Shanghai Jiao Tong University, Shanghai, China; ^2^Shanghai Key Laboratory for Assisted Reproduction and Reproductive Genetics, Shanghai, China; ^3^Animal Laboratory, Renji Hospital, School of Medicine, Shanghai Jiao Tong University, Shanghai, China; ^4^International Peace Maternity and Child Health Hospital, School of Medicine, Shanghai Jiao Tong University, Shanghai, China; ^5^Shanghai Key Laboratory of Embryo Original Diseases, Shanghai, China; ^6^Department of Science and Technology Research, Shanghai Model Organisms, Shanghai, China

**Keywords:** prenatal exposure, estradiol, surrogate fostering, insulin resistance, microbiota-gut-brain axis

## Abstract

**Introduction:**

Prenatal and early postnatal development are known to influence future health. We previously reported that prenatal high estradiol (HE) exposure induces insulin resistance in male mice by disrupting hypothalamus development. Because a foster dam can modify a pup’s gut microbiota and affect its health later in life, we explored whether surrogate fostering could also influence glucose metabolism in HE offspring and examined mechanisms that might be involved.

**Methods:**

We performed a surrogate fostering experiment in mice and examined the relationship between the metabolic markers associated to insulin resistance and the composition of the gut microbiota.

**Results:**

HE pups raised by HE foster dams (HE-HE) developed insulin resistance, but HE pups fostered by negative control dams (NC-HE) did not. The gut microbiota composition of HE-HE mice differed from that of NC mice raised by NC foster dams (NC-NC), whereas the composition in NC-HE mice was similar to that of NC-NC mice. Compared with NC-NC mice, HE-HE mice had decreased levels of fecal short-chain fatty acids and serum intestinal hormones, increased food intake, and increased hypothalamic neuropeptide Y expression. In contrast, none of these indices differed between NC-HE and NC-NC mice. Spearman correlation analysis revealed a significant correlation between the altered gut microbiota composition and the insulin resistance-related metabolic indicators, indicating involvement of the microbiota-gut-brain axis.

**Discussion:**

Our findings suggest that alterations in the early growth environment may prevent fetal-programmed glucose metabolic disorder *via* modulation of the microbiota-gut-brain axis. These findings offer direction for development of translational solutions for adult diseases associated with aberrant microbial communities in early life.

## Introduction

Perinatal development exerts life-long effects on health and disease (Bateson et al., 2004). This observation has provided important clues for the etiological study and prevention of chronic disease, and has contributed to an increased emphasis on prenatal and postnatal care worldwide. With a rising rate in infertility, assisted reproductive technology (ART) has been adopted widely and has been predicted to contribute an extra 400 million (3.5%) people to the global population by 2,100 (Faddy et al., 2018). The potential health effects of ART on the resulting offspring have received increasing attention. We previously reported that glucose metabolism is impaired in male offspring from fresh embryo transfer with supraphysiologic maternal estradiol during early pregnancies generated by ovulation induction (Wang et al., 2018). This maternal hormone-induced metabolic disorder has been attributed to an intrauterine programming effect on hypothalamus development (Wang et al., 2018, 2021). In the current study, we sought to develop a more comprehensive understanding of the underlying mechanisms and to consider feasible approaches to prevention.

Glucose homeostasis is under the control of cross-talk between multiple organs, and the microbiota-gut-brain axis has emerged as a novel mechanism that is gaining attention (Mayer et al., 2015; Grasset and Burcelin, 2019). In this regulatory axis, microbial metabolites stimulate intestinal endocrine cells to produce a series of peptides. These peptides either directly enter the blood circulation or activate immune responses and vagus nerve *via* the enteric nervous system, ultimately affecting hypothalamic neuronal activity to modify metabolic functions (Coll and Yeo, 2013; Mayer et al., 2015). The past decade has seen a proliferation of studies implicating the microbiota-gut-brain axis in conditions such as obesity, diabetes, autism, and neurodegenerative diseases, indicating its significance in chronic disease and life-long health (Cryan et al., 2019). Thus, it is possible that this regulatory axis involves in the insulin resistance that develops after prenatal estradiol exposure.

The gut microbiota is the largest microecosystem of higher organisms, and has a complex and dynamic symbiotic relationship with the host (Heintz-Buschart and Wilmes, 2018). The gut microbiota colonizes prenatally (Walker et al., 2017; Younge et al., 2019) and its composition is shaped by genetic, nutritional, and environmental factors before and after birth (Gomaa, 2020). Alteration of the fostering environment after birth can thus be expected to influence the gut microbiota. Cross-fostering studies indicate that the nursing dam can permanently change the microbiota of foster pups from infancy (Daft et al., 2015; Treichel et al., 2019), and that this may affect health and disease later in life.

Short-chain fatty acids (SCFAs) are the most abundant microbiota-derived metabolites in the gut, mainly including acetate, propionate, and butyrate (Koh et al., 2016). They are involved in glucose metabolism through multiple mechanisms (Canfora et al., 2015; Kim et al., 2018). In the microbiota-gut-brain axis, SCFAs activate intestinal L cells to promote release of peptide YY (PYY) and glucagon-like peptide 1 (GLP-1), which are transported to the hypothalamus and reduce appetite, food intake, and weight gain (Kim et al., 2018; Kjaergaard et al., 2019). SCFAs are thus important for regulating glucose homeostasis.

Here, we examined whether the gut microbiota composition and the microbiota-gut-brain axis play a role in prenatal estradiol-induced adult hypothalamic insulin resistance (Wang et al., 2018) by conducting a surrogate foster experiment. This study will advance our understanding of prenatally derived glucose metabolism disorders and provide fresh insights into early intervention approaches.

## Materials and methods

### Animal model

We employed a previously described mouse model of prenatal high estradiol (HE) exposure (Wang et al., 2018). Briefly, 8-week-old pregnant C57BL/6 mice received 100 μg/kg/d estradiol valerate (Sigma) by gavage from E5.5 to E11.5 in the HE group, or an equal volume of solvent (corn oil) in the negative control (NC) group. Only male pups were included in the analysis (Wang et al., 2018). Newborn male mice were fostered by non-birth mothers who had given birth in the past 48 h and were ready to nurse, together with their biological offspring. The combinations of nursing mothers and foster pups were as follows: NC nursing mother with NC foster pup (NC-NC), HE nursing mother with HE foster pup (HE-HE), and NC nursing mother with HE foster pups (NC-HE). The number of foster pups and biological pups for each dam was 2: 2, and the foster pups in each given dam were littermates. Fostered pups were weaned at the age of 3 weeks and housed individually until the end of the experiment at 24 weeks. All mice were provided with the same sterilized food and wood chip bedding, and all operations were performed on a clean bench.

### Glucose and insulin tolerance tests

Intraperitoneal glucose tolerance tests (GTTs) and insulin tolerance tests (ITTs) were performed at 3 and 8 weeks after birth as described previously (Wang et al., 2018). Mice were fasted overnight for 16 h before being injected intraperitoneally with glucose at 2 g/kg body weight for the GTT, and were fasted for 6 h before being injected intraperitoneally with insulin at 1 U/kg body weight for the ITT. Glucose levels in tail blood were measured using an automatic glucometer (Roche) at 0, 30, 60, and 120 min after glucose or insulin injection. The area under the curve (AUC) was calculated to measure glucose and insulin tolerance.

### Food intake

The daily intake of each mouse was determined as described previously (Wang et al., 2018) as the difference between the weight of food given and the weight remaining after 24 h. Daily intake was monitored for 1 week and the average was calculated.

### Enzyme-linked immunosorbent assay

Mice were anaesthetized with isoflurane and blood was collected by removing the eyeball. Serum levels of insulin, GLP-1, PYY, and cholecystokinin (CCK) were analyzed using ELISA kits from Crystal Chem (insulin) and Cusabio (GLP-1, PYY, and CCK) according to the manufacturer’s instructions. In brief, serum samples and standards were incubated in the microtiter plate wells, followed by incubation with conjugate solution, substrate solution, and stop solution. The optical density values were measured at 450 nm using a microplate reader. A standard curve is constructed to determine the concentrations of target protein.

### Tissue immunofluorescence

Mice were transcardially perfused with 4% paraformaldehyde under anesthesia. Brains were removed and fixed in 4% paraformaldehyde for 4 h and then infiltrated with 20 to 30% sucrose. Brain sections (20 μm) were made using a freezing microtome (Leica), blocked with 5% bovine serum albumin/0.3% Triton X-100 for 1 h at room temperature, and incubated with primary antibodies overnight at 4°C. Sections were reacted with secondary antibodies at room temperature for 2 h and counterstained with 4′,6-diamidino-2-phenylindole. Primary antibodies were rabbit anti–neuropeptide Y (NPY) (1: 800, Cell Signaling Technology, catalog no. 11976) and rabbit anti-proopiomelanocortin (POMC) (1: 500, Abcam, catalog no. ab254257). The secondary antibody was anti-rabbit Alexa Fluor 594 (1: 1000, Abcam, catalog no. ab150080). NPY-and POMC-positive cells were counted in five serial sections from each mouse using ImageJ software (National Institutes of Health; Arena et al., 2017).

### Quantitative real-time polymerase chain reaction

Mice were euthanized by decapitation, brains were quickly removed, and hypothalami were dissected and homogenized in TRIzol (Invitrogen) on ice. RNA extraction and qPCR were performed to detect the expression of *Npy* and *Pomc* as previously described (Wang et al., 2021). The primers used are listed in Table 1, and β-actin was used as an endogenous control. Relative expression was determined using the 2^–ΔΔCT^ method.

**Table 1:** Primers used for qPCR.

Gene	Primer sequence
*β-actin*	Forward 5′-GTCCCTCACCCTCCCAAAAG-3′
	Reverse 5′-GCTGCCTCAACACCTCAACCC-3′
*Npy*	Forward 5′-ATGCTAGGTAACAAGCGAATGG-3′
	Reverse 5′-TGTCGCAGAGCGGAGTAGTAT-3′
*Pomc*	Forward 5′-ATGCCGAGATTCTGCTACAGT-3′
	Reverse 5′-TCCAGCGAGAGGTCGAGTTT-3′

### Microbiota community analysis

Fresh feces of offspring were collected with sterile equipment after stimulating the perianal area. Vaginal lavage fluid of pregnant mice was collected by flushing the vagina with 20 μl sterile phosphate-buffered saline using a sterile pipette. We performed 16S rRNA sequencing to analyze the microbiota composition. Total DNA of fecal and vaginal lavage samples was extracted using the QIAamp Rapid DNA Mini kit (Qiagen), and a sequencing library was constructed from amplicons targeting the V3 and V4 regions of the 16S ribosomal RNA gene using primers 338F (5′-ACTCCTACGGGAGGCAGCAG-3′) and 806R (5′-GGACTACHVGGGTWTCTAAT-3′). Paired-end sequencing was performed on the Illumina MiSeq platform, and raw data were filtered by Trimmomatic and FLASH software. All clean reads were clustered into operational taxonomic units using Ribosomal Database Project (RDP) Classifier (v. 2.11) at 97% sequence similarity. Taxonomic assignment of operational taxonomic units was performed using the RDP database (Cole et al., 2014). Chao 1 and Shannon indices were calculated using QIIME 2 (version 2020.2) (Caporaso et al., 2010), and principal coordinates analysis (PCoA) was conducted based on the weighted UniFrac distance. PICRUST2 was used to predict the functional composition of bacterial genera based on the Kyoto Encyclopedia of Genes and Genomes (KEGG) database (Douglas et al., 2020).

### SCFA analysis

SCFAs were measured by liquid chromatography–tandem mass spectrometry (LC–MS/MS) through a commercial laboratory (Lumingbio, Shanghai, China). Briefly, fecal and serum samples were homogenized and ultrasound-extracted in 50% (v/v) acetonitrile/water containing an isotopic internal standard. After centrifugation, supernatants were derivatized with 200 mM 3-nitrophenylhydrazine and 120 mM ethylcarbodiimide hydrochloride-6% pyridine for 30 min at 40°C. Reaction mixtures were analyzed on an AB ExionLC/AB Sciex Qtrap 6500+ LC–MS/MS system.

### Statistical analysis

A one-way analysis of variance (ANOVA) with Tukey *post hoc* test was used for comparisons among three groups, and an unpaired Student’s *t*-test was used for comparisons between two groups using the Statistical Package for Sciences Software, v. 21.0 (IBM). Data are presented as mean ± standard error of the mean (SEM). Spearman correlation analysis was performed and plotted using R software. *p* < 0.05 was considered statistically significant.

## Results

### Surrogate fostering prevents insulin resistance in mice exposed prenatally to high estradiol

**Figure 1:**
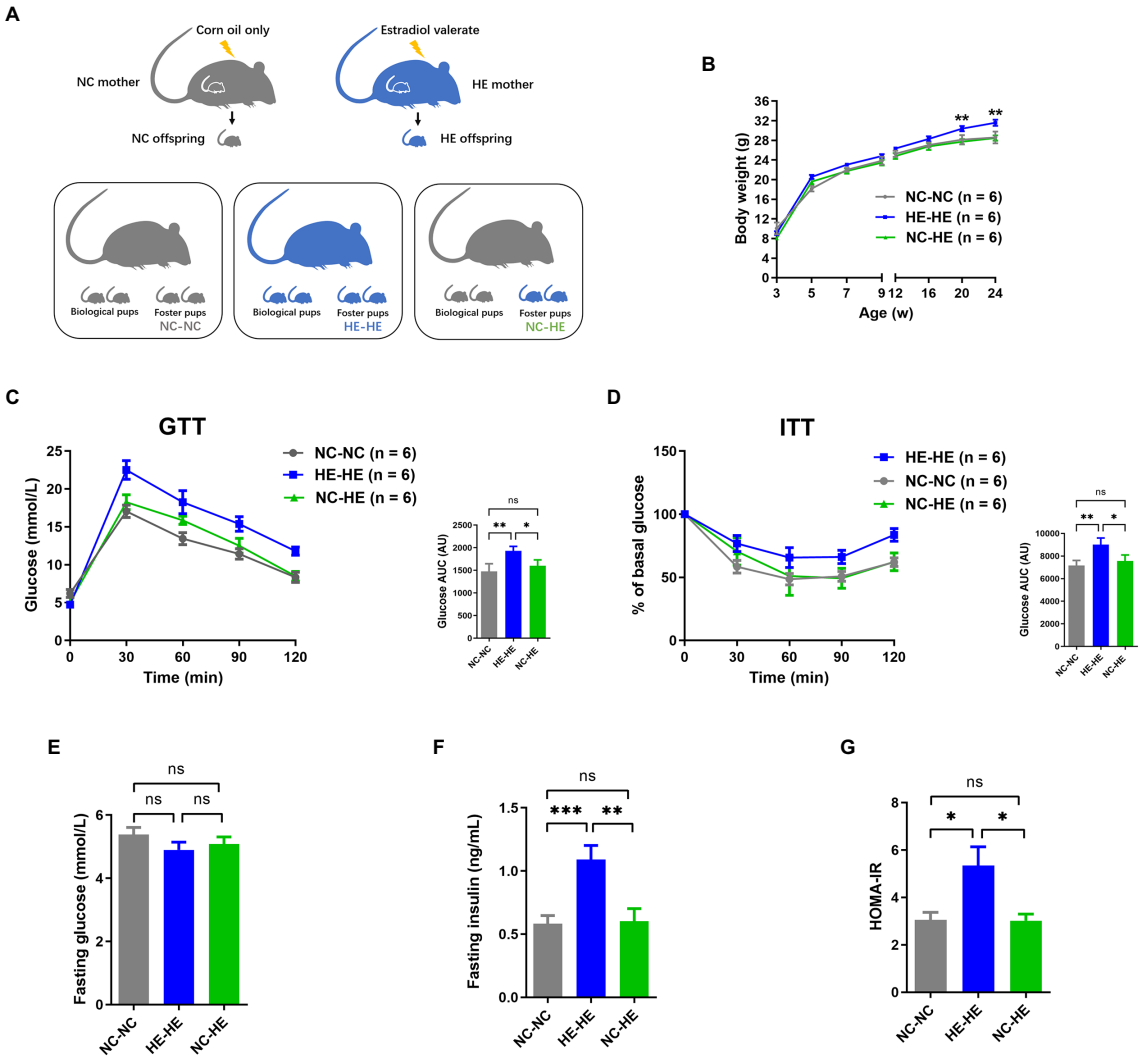
Body weight and glucose metabolism assessments of NC-NC, HE-HE, and NC-HE mice from the surrogate fostering mouse model. **(A)** Schematic of method used to generate the model. **(B)** Body weight from 3 to 24 weeks after birth. **(C)** GTT, **(D)** ITT, **(E)** fasting glucose, **(F)** fasting insulin, and **(G)** HOMA-IR scores at 24 weeks after birth. In **(C,D)**, left shows raw data and right shows AUC in arbitrary units (AU). Error bars represent SEM. Significance determined by one-way ANOVA. *p < 0.05; **p < 0.01; ***p < 0.001; ns, not significant.

To induce the mouse model of prenatal HE exposure (Wang et al., 2018), pregnant 8-week-old C57BL/6 mice received gavage of estradiol valerate in corn oil (HE) or corn oil alone (negative control or NC). We compared male HE pups fostered by another HE dam (HE-HE) to male HE pups fostered by an NC dam (HE-NC) and male NC pups fostered by another NC dam (NC-NC) (Figure 1A).

To minimize the influence of handling on the odor of fostered pups and thus avoid cub eating, we monitored pup body weight from weaning at 3 weeks rather than from birth. Consistent with our previous study (Wang et al., 2018), the weight of HE-HE male mice exceeded that of the NC-NC group beginning at 20 weeks; in contrast, no significant difference was observed between the NC-HE and NC-NC groups (Figure 1B). Based on our previous observation that male HE mice present insulin resistance at 24 weeks (Wang et al., 2018), we examined glucose metabolism in the three foster groups at this age. The AUC for both GTT and ITT was higher in the HE-HE group, with no significant difference between the NC-HE and NC-NC groups (Figures 1C,D). We also compared fasting glucose, fasting insulin, and homeostasis model assessment for insulin resistance (HOMA-IR) scores at 24 weeks. No differences in fasting glucose were observed among the three groups (Figure 1E). On the other hand, fasting insulin and HOMA-IR scores were increased in HE-HE mice, with no difference between the NC-HE and NC-NC groups (Figures 1F,G). These observations indicated that HE pups fostered by NC dams no longer presented insulin resistance in later life as those fostered by HE dams did.

### Surrogate fostering modulates the composition of gut microbiota in male HE mice

**Figure 2:**
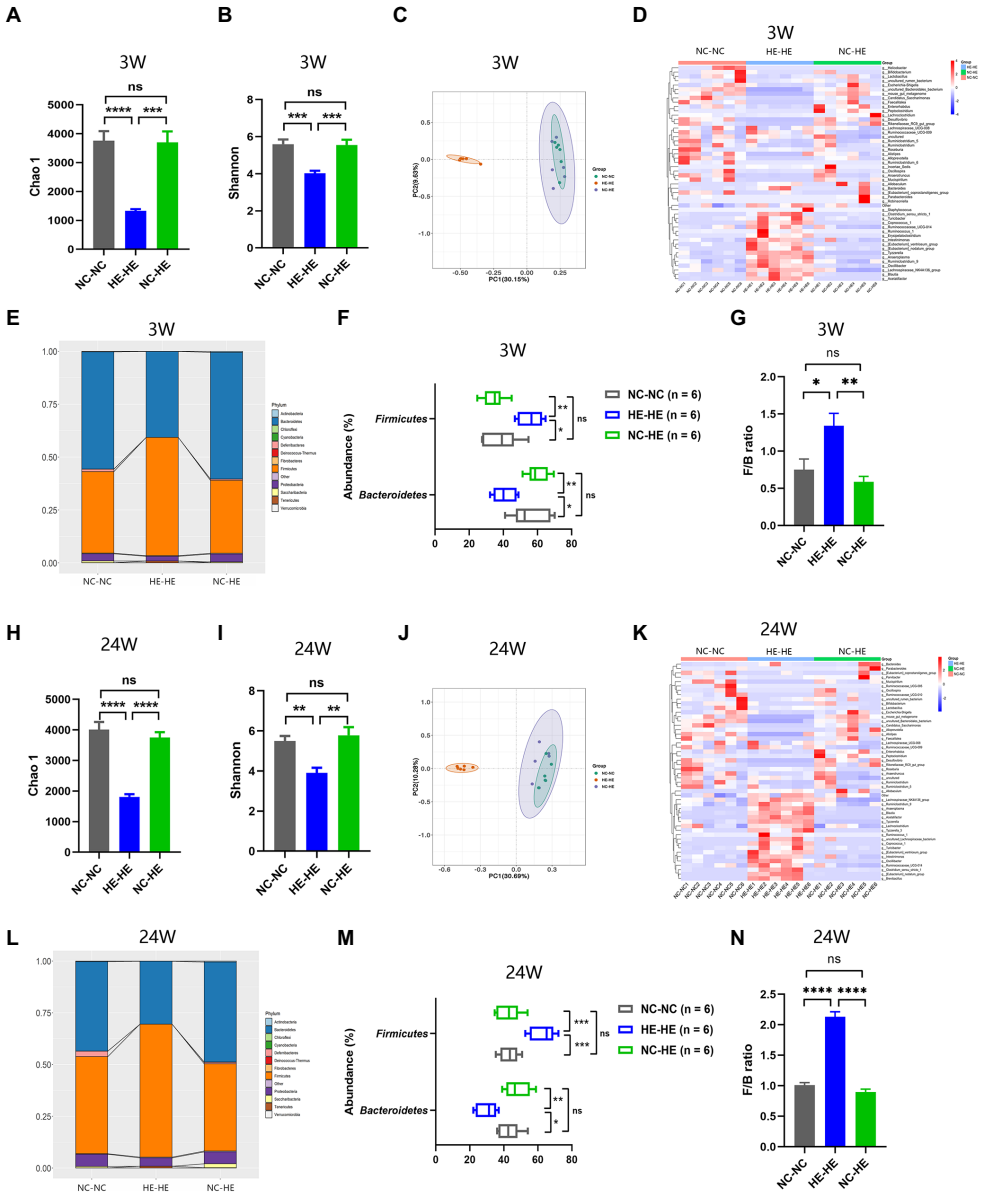
Gut microbiota composition analysis in 3-week-old **(A–G)** and 24-week-old **(H–N)** NC-NC, HE-HE, and NC-HE mice. **(A,H)** Chao 1 index, **(B,I)** Shannon index, and **(C,J)** PCoA plots of gut microbiota. **(D,K)** Heatmap of top 50 differentiated genera. **(E,L)** Phylum distribution. **(F,M)** Abundance of *Firmicutes* and *Bacteroidetes*. **(G,N)** Relative *Firmicutes*/*Bacteroidetes (F/B)* ratio. Error bars represent SEM. Significance determined by one-way ANOVA. *p < 0.05; **p < 0.01; ***p < 0.001; ****p < 0.0001; ns, not significant.

Because the nursing dam has been shown to induce lasting alterations in the microbiota of foster pups (Daft et al., 2015; Treichel et al., 2019), we sought to investigate whether the prevention of insulin resistance in NC-HE mice could be attributed to a correction of a microflora disorder. We performed 16S rRNA sequencing to analyze the microbiota composition of the intestinal contents in the three groups at 3 and 24 weeks. Chao 1 and Shannon indices were used as measures of α diversity, and PCoA plots were used for β diversity. At both 3 and 24 weeks, HE-HE mice had decreased α diversity, with no significant difference between the NC-NC and NC-HE groups (Figures 2A,B, H, I). The HE-HE clusters in the PCoA plots were clearly separate from the NC-NC and NC-HE clusters, which were overlapped (Figures 2C, J), indicating that there was no significant difference between NC-NC and NC-HE. The heatmap of the top 50 differentiated taxa with the highest genus level at both 3 and 24 weeks demonstrated that the microbiota community composition in HE-HE mice was distinct from that of the other two groups, and that the NC-HE group was closer to the NC-NC group (Figures 2D, K). Phylum-level analysis revealed that the taxonomic distribution of the HE-HE group differed markedly from that of the NC-NC and NC-HE groups (Figures 2E, L), with a significantly higher abundance of *Firmicutes* and lower abundance of *Bacteroidetes* (Figures 2F, M), leading to an increased ratio of *Firmicutes*/*Bacteroidetes* (F/B) (Figures 2G, N). The NC-NC and NC-HE samples had no significant difference in phylum distribution or F/B ratio. These results indicate that HE exposure leads to a change in gut microbiota composition prior to weaning that is sustained at least until the observed age of insulin resistance, and that surrogate fostering by an NC dam prevents this change in gut microbiota.

### Surrogate fostering restores fecal SCFA and intestinal hormone levels in male HE mice

**Figure 3:**
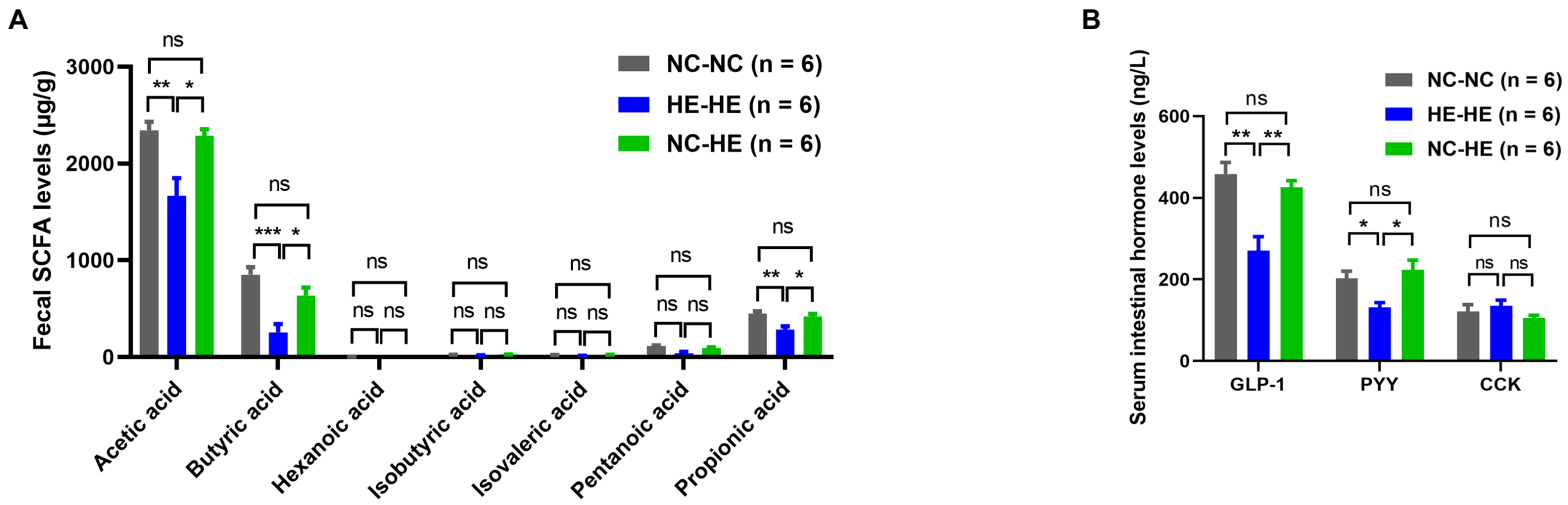
Fecal SCFA **(A)** and serum intestinal hormone **(B)** analysis in 24-week-old NC-NC, HE-HE, and NC-HE mice. Error bars represent SEM. Significance determined by one-way ANOVA. *p < 0.05; **p < 0.01; ***p < 0.001; ns, not significant.

To further investigate how surrogate fostering affects metabolism, we measured gut microbiota metabolites in feces at 24 weeks using LC–MS/MS. Acetic acid, butyric acid, and propionic acid concentrations all decreased significantly in the HE-HE group and were restored in the NC-HE group (Figure 3A). Other SCFAs were not significantly changed. In addition, as downstream molecules of SCFAs in blood circulation, we assessed the levels of serum intestinal hormones GLP-1, PYY, and CCK by ELSIA. GLP-1 and PYY were decreased in HE-HE mice and were restored in NC-HE mice (Figure 3B). CCK levels did not differ among the three groups.

### Surrogate fostering attenuates increased food intake and hypothalamic NPY in male HE mice

**Figure 4:**
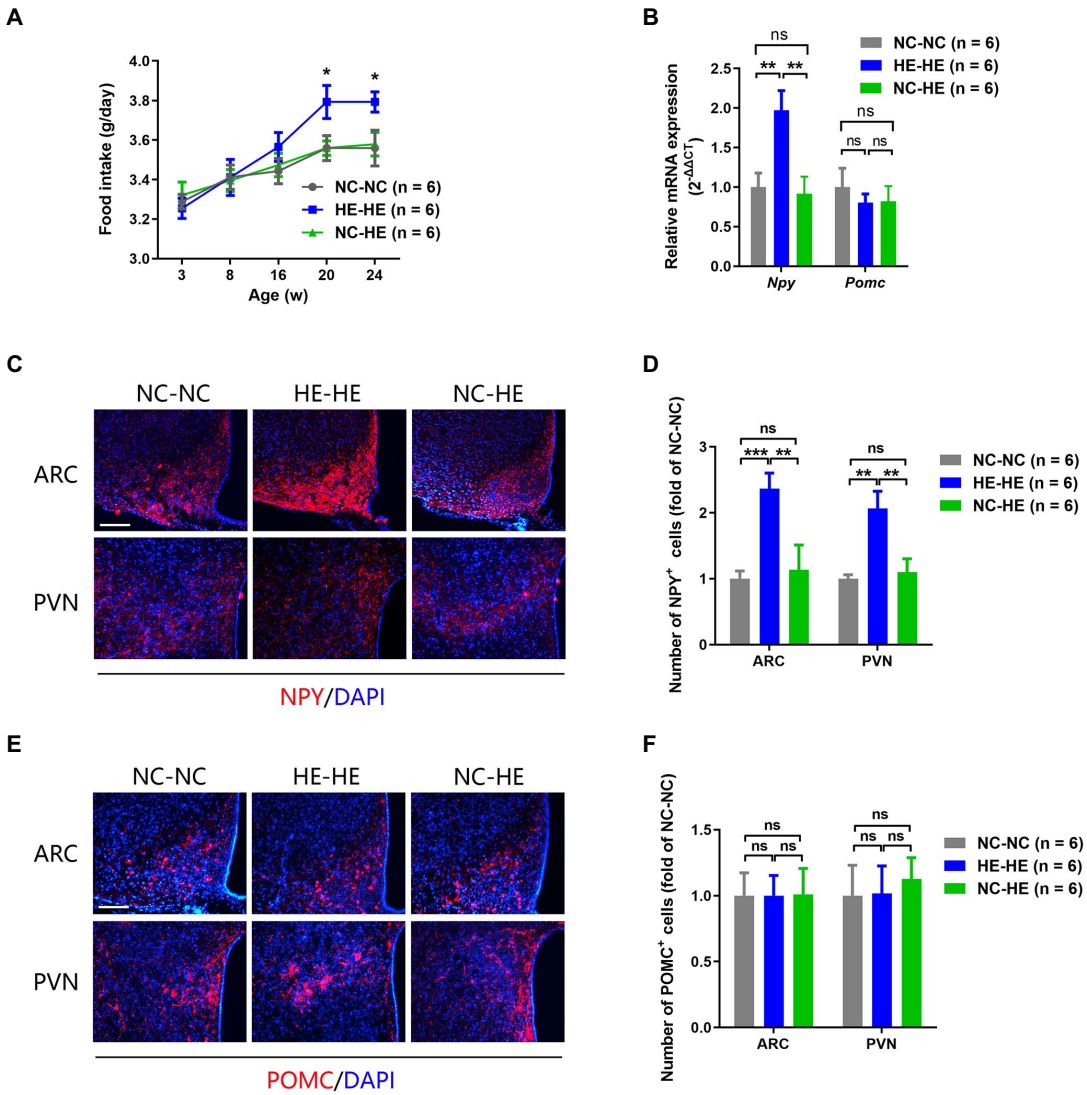
Food intake and hypothalamic neuropeptide expression in NC-NC, HE-HE, and NC-HE mice. **(A)** Daily food intake at 3–24 weeks. **(B)** qPCR of hypothalamic *Npy* and *Pomc* at 24 weeks of age. **(C,E)** Representative images of NPY and POMC immunolabeling in the arcuate nucleus (ARC) and paraventricular nucleus (PVN). Scale bars: 100 μm. **(D,F)** Quantification of NYP-and POMC-positive cells in the ARC and PVN. Error bars represent SEM. Significance determined by one-way ANOVA. *p < 0.05; **p < 0.01; ***p < 0.001; ns, not significant.

Prenatal HE exposure increases both food intake and hypothalamic NPY expression, which contribute to weight gain and insulin resistance (Wang et al., 2018), so we examined these parameters in fostered mice. Food intake in HE-HE mice began to increase at 8 weeks, significantly exceeded that of NC-NC and NC-HE mice at 20 weeks, but there was no a difference in food intake between NC-NC and NC-HE mice (Figure 4A). In all three groups, food intake increased with body weight (Figure 1B). Hypothalamic NPY (orexigenic) and POMC (anorexigenic) were examined because they play significant roles in stimulating and suppressing appetite, respectively. Results of qPCR revealed significantly increased expression of *Npy* mRNA in HE-HE mice at 24 weeks, but no difference between NC-HE and NC-NC mice (Figure 4B). *Pomc* mRNA expression did not differ among the three groups (Figure 4B). Immunostaining revealed a significant increase in NPY-positive cells in the arcuate paraventricular nuclei of HE-HE hypothalami, but no difference between NC-HE and NC-NC hypothalami (Figures 4C,D). The number of POMC-positive cells was similar among all three groups (Figures 4E,F).

### Correction of insulin resistance by surrogate fostering involves the microbiota-gut-brain axis

**Figure 5:**
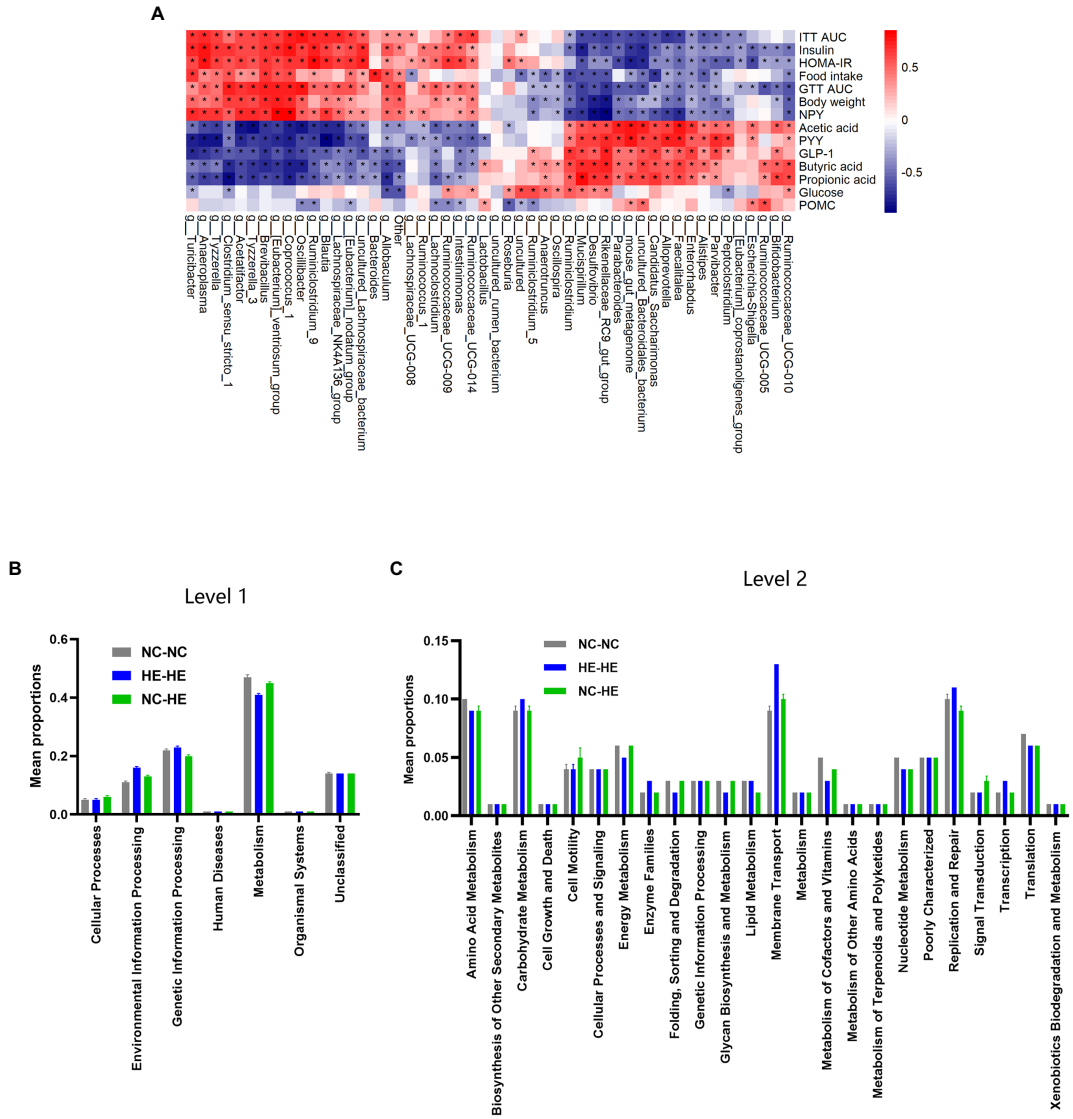
Correlation analysis between gut bacterial genera and metabolic indices. **(A)** Heatmap of Spearman correlation analysis between the top 50 differentiated genera and metabolic indices. The degree of correlation is shown by a gradient from red (positive correlation) to blue (negative correlation); asterisks indicate *p* < 0.05. **(B,C)** KEGG pathway prediction of genera at levels 1 **(B)** and 2 **(C)** by PICRUSt2 analysis.

We next assessed the association between the changes in gut microbiota composition and the metabolic alterations observed at 24 weeks. Spearman correlation analysis was performed between the top 50 enriched genera and the significantly different metabolic indices among the three groups. For metabolic changes, we included acetic acid, butyric acid, propionic acid, GLP-1, and PYY as beneficial indices, and GTT AUC, ITT AUC, fasting insulin, HOMA-IR score, food intake, and body weight as adverse indices. In Figure 5A, correlation index values are presented in a heatmap with asterisks to indicate a significance of *p*<0.05. The majority of genera showed opposite correlations between beneficial and adverse indices, and 22 of 50 presented a significant correlation with all metabolic indices.

**Figure 6:**
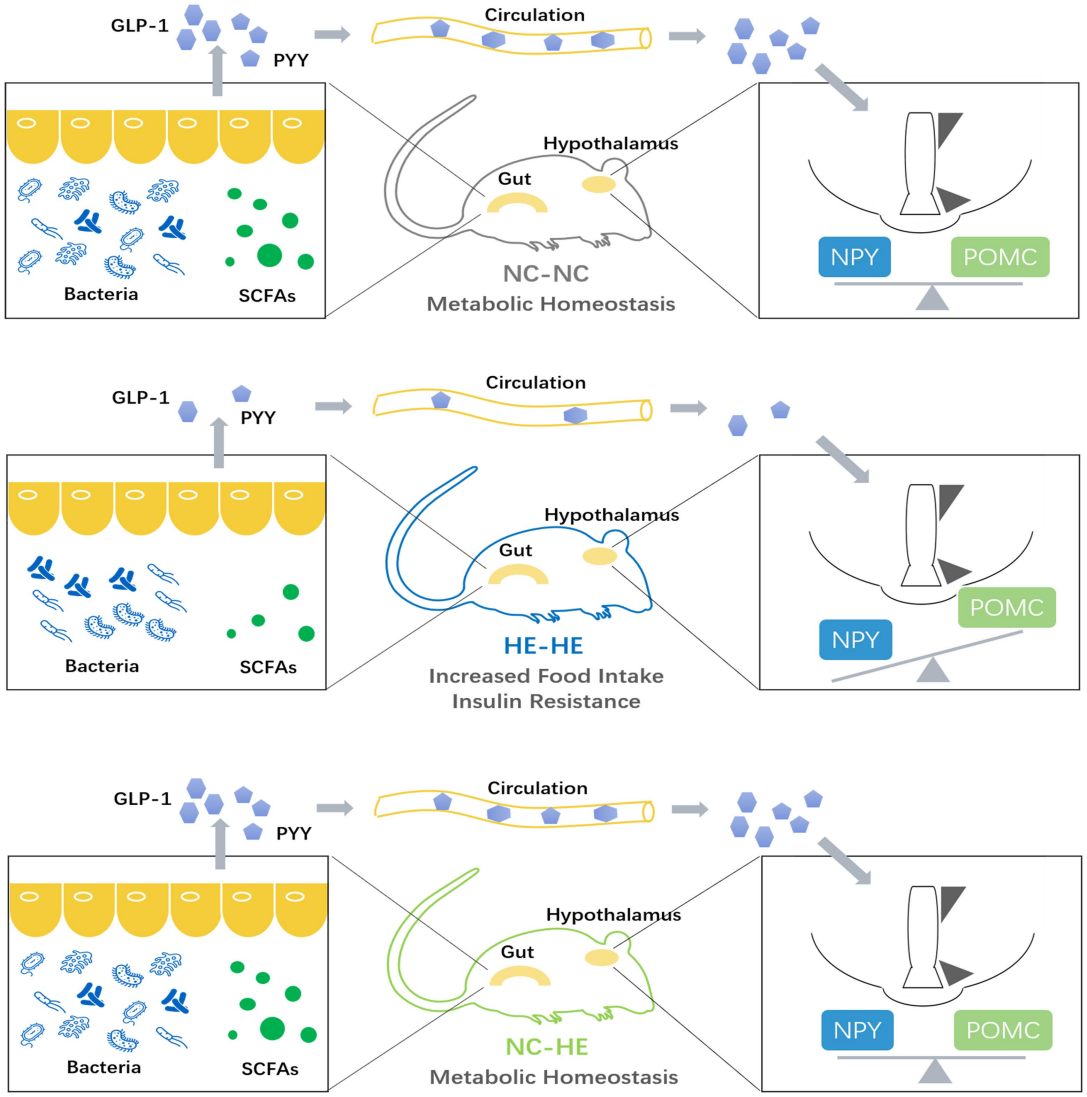
Model illustrating the role of the microbiota-gut-brain axis in the prevention of prenatal high estradiol-induced insulin resistance by surrogate fostering.

Finally, we used PICRUSt2 software online^1^ to predict the functional composition of these 22 genera based on the KEGG database. The results indicate that metabolism was the most abundant pathway (level 1), accounting for 41–47% of the total relative abundance (Figure 5B). At level 2, the main contributors to metabolism were carbohydrate metabolism (9.1–9.6%), amino acid metabolism (8.6–9.5%) and energy metabolism (4.5–6.4%) (Figure 5C). Therefore, the altered gut microbiota composition resulting from surrogate fostering correlated significantly with the correction of insulin resistance, fecal SCFAs, serum intestinal hormones, and hypothalamic NPY. These factors constitute a microbiota-gut-brain axis that exerted a remodeling effect on prenatal HE-induced glucose metabolic disorder (Figure 6).

## Discussion

Prenatal and postnatal development are critical in the etiology of many chronic diseases. An adverse intrauterine environment contributes to the risk of pathologic conditions such as metabolic syndrome, cardiovascular diseases, and behavioral or cognitive dysfunctions, leading to impaired quality of life and shortened life span (Drever et al., 2010; Rinaudo and Wang, 2012; Reynolds et al., 2019). As ART is increasingly adopted worldwide, involving conditions of abnormal prenatal hormone exposure, monitoring the life-long health of the resulting offspring and exploring interventions for proven adverse outcomes will have undeniable benefits for global health.

Supraphysiologic maternal estradiol induced glucose and insulin intolerance in male rather than female human offspring, and this sex-specific effect was confirmed in a mouse model (Wang et al., 2018). Therefore, as a continuation and extension of this previously published research, our current study continues to focus on male pups. We hypothesize that this gender disparity is caused by male offspring’s susceptibility to prenatal high estradiol exposure, albeit the precise mechanism needs to be studied further.

We demonstrated here that prenatal high estradiol exposure disrupted the gut microbiota composition in mice, and that these changes were closely related to changes in metabolic function. It is not clear whether maternal estradiol affects the offspring’s gut microbiota directly in the uterus or indirectly after birth. In the current literature, there is no consistent evidence of bacterial communities in mouse fetal tissue (Theis et al., 2020), and cultivatable bacteria with low abundance have been detected in the mouse fetal intestine during mid-gestation but not late gestation (Younge et al., 2019). This suggests that estradiol exposure may not have a direct effect on the microbiota *in utero*, and also that the fetal microbiota is less likely than the postnatal microbiota to affect later health. Since we also discovered that high maternal estradiol modulates hypothalamic neurogenesis and induces hypothalamic insulin resistance in offspring, it is reasonable to speculate that bidirectional brain-gut communication may play a role in changing the gut microbiota in HE-HE mice after birth (Martin et al., 2018), but further investigation is required to elucidate the specific mechanism(s) involved.

Early development is highly vulnerable to environmental challenges, but mounting evidence suggests that favorable conditions can revert the detrimental outcomes. Postnatal antibiotic treatments, diet, and environmental exposures can modulate the infant’s microbiome (Tamburini et al., 2016). As a natural source of microbes, the rearing environment plays a crucial role in bacterial colonization in newborns. Cohabitation is known to increase bacterial exchange (Flores et al., 2014), and family members have more similar microbiota than unrelated individuals (Song et al., 2013). Therefore, the idea of dam-to-pup microbiome transfer in the surrogate fostering experiment is valid and feasible.

Because fostering by a nursing mother most certainly induces stress in pups, we designed the experiment so that pups in all groups are raised by foster mothers. The effect of stress on the microbiota is insignificant compared with the effect of the nursing dam itself (Treichel et al., 2019). We compared the vaginal microbiota in HE and NC mothers before delivery at E18.5. The genus heatmap presented a similar microbiota composition (Supplementary Figure S1), ruling out the possibility that the altered gut microbiome in the HE offspring originated from the maternal vagina. This finding supports our previous result that an increased risk of insulin resistance developed in male newborns and children of fresh embryo transfer (exposed to high maternal estradiol) born both by cesarean delivery and by vaginal delivery (Wang et al., 2018). Based on these observations, we speculate that the different microbiota transferred from dam to pup in our experiment is mainly attributable to a direct transfer through breast milk, or exposure to skin, saliva, or feces of the nursing mother or the co-reared biological pups. Further studies are needed to address this question. Likewise, human studies have verified that the bacteria are vertically transferred from mother to infant by breast feeding (Pannaraj et al., 2017; Wang S. et al., 2020), they can also be determined by older siblings in the home (Christensen et al., 2022). Other perinatal conditions like type of feeding, lifestyle, and environmental exposure can also modulate the development and maturation of the infant gut microbiota in human children (Derrien et al., 2019). These evidences support the supposed way of microbiota transfer in our experiment discussed above.

We found that HE-HE mice had a distinctly different fecal microbiota composition at weaning and in adulthood. This was observed as changes in α diversity, β diversity, and taxonomic distribution. The reduced microbial diversity and elevated F/B ratio in HE-HE pups was consistent with the insulin resistance phenotype (Pascale et al., 2019). The 3-week and 24-week genus heatmaps shared decreased abundances of *Alistipes* and *Alloprevotella* and increased abundances of *Anaerotruncus, Oscillibacter, Blautia, Allobaculum, Acetatifactor, Turicibacter, Tyzzerella,* and *Intestinimonas* in HE-HE feces (Figures 2D, K). These changes were reversed when HE pups were fostered by NC dams. According to published studies, a drop in *Alloprevotella* (Wang J. et al., 2020; Wu et al., 2021; Zhang et al., 2021; Zhao et al., 2021; Hao et al., 2022; Liu et al., 2022) and an increase in *Blautia* (Zhao et al., 2021; Bao et al., 2022) and *Tyzzerella* (Li et al., 2022) are found in animal models of in insulin resistance or glucose intolerance. On the other hand, antidiabetic medications and bioactive compounds modulate the gut microbial community of diabetic mouse model, characterized by increased *Alistipes* (Hu et al., 2019; Jeong et al., 2021; Li et al., 2021; Wu R. et al., 2022) and decreased *Anaerotruncus* (Yong et al., 2022), *Oscillibacter* (Lin et al., 2022; Wang et al., 2022), *Allobaculum* (Jia et al., 2017; Shen et al., 2021; Xu et al., 2021; Ma et al., 2022), *Acetatifactor* (Wu Y. et al., 2022) and *Turicibacter* (Zhao et al., 2021). These findings coincide with the correlation between microbiota and metabolic phenotypes in our study. However, other studies claim *Alistipes* positively correlates with glucose intolerance (Han et al., 2020; Li et al., 2022), while *Allobaculum* (Li et al., 2017; Wang et al., 2017), *Acetatifactor* (Zhao et al., 2022) and *Intestinimonas* (Cai et al., 2020; Ma et al., 2022) are beneficial genera in glucose metabolism. It should be noted that a recent study warned about the inconsistency of microbe-disease associations across a large number of public cohorts and individuals (Tierney et al., 2022). Therefore, our study should be valued for its demonstration of an insulin resistance correction *via* modulation of the overall microbiota community rather than as an etiologic mechanism involving any specific bacterial genus or genera.

Gut-derived SCFAs are microbiota-produced fermentation products. They exert beneficial properties including improving insulin sensitivity, inhibiting white adipose tissue accumulation, and suppressing inflammation (Bolognini et al., 2021). Because over 95% of SCFAs produced in the gut are absorbed by the host (den Besten et al., 2013), the decreased fecal SCFAs observed in HE-HE mice could be attributed to either decreased production or increased absorption. We therefore checked blood levels of acetic acid, butyric acid, and propionic acid and found that they were also decreased in HE-HE mice (Supplementary Figure S2), indicating a reduced total production. This could be associated with the reduced diversity of gut microbes (Martin-Gallausiaux et al., 2021) and the decreased abundance of SCFA-producers like *Alistipes* (Parker et al., 2020) and *Alloprevotella* (Chen et al., 2020) as we described above.

The hypothalamic arcuate nucleus–paraventricular nucleus feeding network releases NPY and POMC, playing a major role in feeding regulation and hypothalamus-centered glucoregulatory system (Parker and Bloom, 2012). We found that hypothalamic NPY was elevated in HE-HE mice and restored in NC-HE mice, while the POMC levels were unaffected by the foster dam. This indicates that the metabolic effect of the nursing dam was exerted through inhibition of orexigenic neurons rather than activation of anorexigenic neurons. Gut microbes produce other neuroactive substances such as folate, serotonin, dopamine, and γ-aminobutyric acid (Asano et al., 2012). The hypothalamus regulates feeding behavior by integrating these interoceptive signals from gut microbes (Moura-Assis et al., 2021) *via* various mechanisms that may involve neurotransmission, neurogenesis, and neuroinflammation (Cryan et al., 2019). Future investigations can examine whether these regulatory mechanisms are also involved in estradiol-induced insulin resistance and its recovery by surrogate fostering.

In our correlation analysis between microbiota and metabolic indices, 5 of the 11 bacterial genera correlated with beneficial metabolic indices belonged to *Bacteroidetes*, and 10 out of the 11 bacterial genera correlated with adverse metabolic indices belonged to *Firmicutes*. PICRUSt2 analysis revealed metabolic pathways as the most enriched function among these 22 genera, and more specifically carbohydrate, amino acid, and energy metabolism. Carbohydrate metabolism includes active carbon-related metabolic pathways and organic substance biotransformation (Wang P. et al., 2020); amino acid metabolism reflects the role of amino acids as energy sources for microbial growth (Lopez-Gonzalez et al., 2015); and energy metabolism is closely related to glucose homeostasis and energy expenditure in the host (Cani et al., 2019). Thus, these functional predictions provide further evidence for the effect of the dam-to-pup transfer of the microbiome. Although the precise effect of these bacterial genera on host metabolism changes in our study has yet to be determined, microbiota analysis combined with metabolomic and proteomic profiling is likely to reveal a more comprehensive microbiota-gut-brain regulatory network.

Taken together, our observations suggest that altering the early growth environment may be a novel prevention and treatment strategy for regulating the gut microbiota, preventing insulin resistance, and correcting other metabolic disorders resulting from intrauterine hormone exposure. These insights may shed light on potential interventions for children prenatally exposed to high estradiol levels. For example, early environmental modifications such as more frequent contact with children without prenatal high estradiol exposure may improve glucose metabolism in their later life. Such hypotheses will require validation by cohort studies. Overall, our research presents a microbiota-gut-brain regulation axis in prenatal estradiol-induced insulin resistance and offers direction for development of translational solutions for adult diseases associated with aberrant microbial communities in early life.

## Conclusion

Our experiments demonstrate that insulin resistance in male mice prenatally exposed to high estradiol can be prevented by surrogate fostering from birth, and that this effect is mediated by a microbiota-gut-brain modulation axis. This study reveals the crucial importance of the postnatal rearing environment in adult health, and sheds new light on possibilities for early prevention of developmentally mediated glucose metabolism disorder.

## Data availability statement

The datasets presented in this study can be found in online repositories. The names of the repository/repositories and accession number(s) can be found at: https://www.ncbi.nlm.nih.gov/bioproject/PRJNA862308.

## Ethics statement

The animal study was reviewed and approved by the Institutional Animal Care and Use Committee of Shanghai Jiao Tong University.

## Author contributions

HW and YS conceived and designed the experiments. HW, CZ, and SG performed the experiments, collected and analyzed the data, and drafted the manuscript. YS revised the final manuscript. All authors reviewed and approved the final version prior to submission.

## Funding

This work was supported by the National Key R&D Program of China (2019YFA0802604), the National Natural Science Foundation of China (81901552 and 82130046), and the Innovative Research Team of High-Level Local Universities in Shanghai (SHSMU-ZLCX20210200 and SSMU-ZLCX20180401).

## Conflict of interest

The authors declare that the research was conducted in the absence of any commercial or financial relationships that could be construed as a potential conflict of interest.

## Publisher’s note

All claims expressed in this article are solely those of the authors and do not necessarily represent those of their affiliated organizations, or those of the publisher, the editors and the reviewers. Any product that may be evaluated in this article, or claim that may be made by its manufacturer, is not guaranteed or endorsed by the publisher.

## Footnotes

^1^ https://www.omicstudio.cn/analysis

## Supplementary material

Supplementary Figure S1 Vaginal microbiota analysis in pregnant E18.5 HE and NC mice. **(A,B)** Chao 1 and Shannon indices (*n* = 3). **(C)** Heatmap of top 30 enriched genera. Error bars represent SEM. Significance determined by Student’s *t*-test. ns, not significant.

Supplementary Figure S2 Serum SCFA concentration in 24-week-old NC-NC, HE-HE, and NC-HE mice. Error bars represent SEM. Significance determined by one-way ANOVA. ***p* < 0.01; ****p* < 0.001; *****p* < 0.0001; ns, not significant.
