# Supplementary figures and images for: Surrogate fostering of mice prevents prenatal estradiol-induced insulin resistance via modulation of the microbiota-gut-brain axis
Source: Front Microbiol. 2023 Jan 9;13:1050352. doi: 10.3389/fmicb.2022.1050352 (PMC9868306; doi:10.3389/fmicb.2022.1050352)

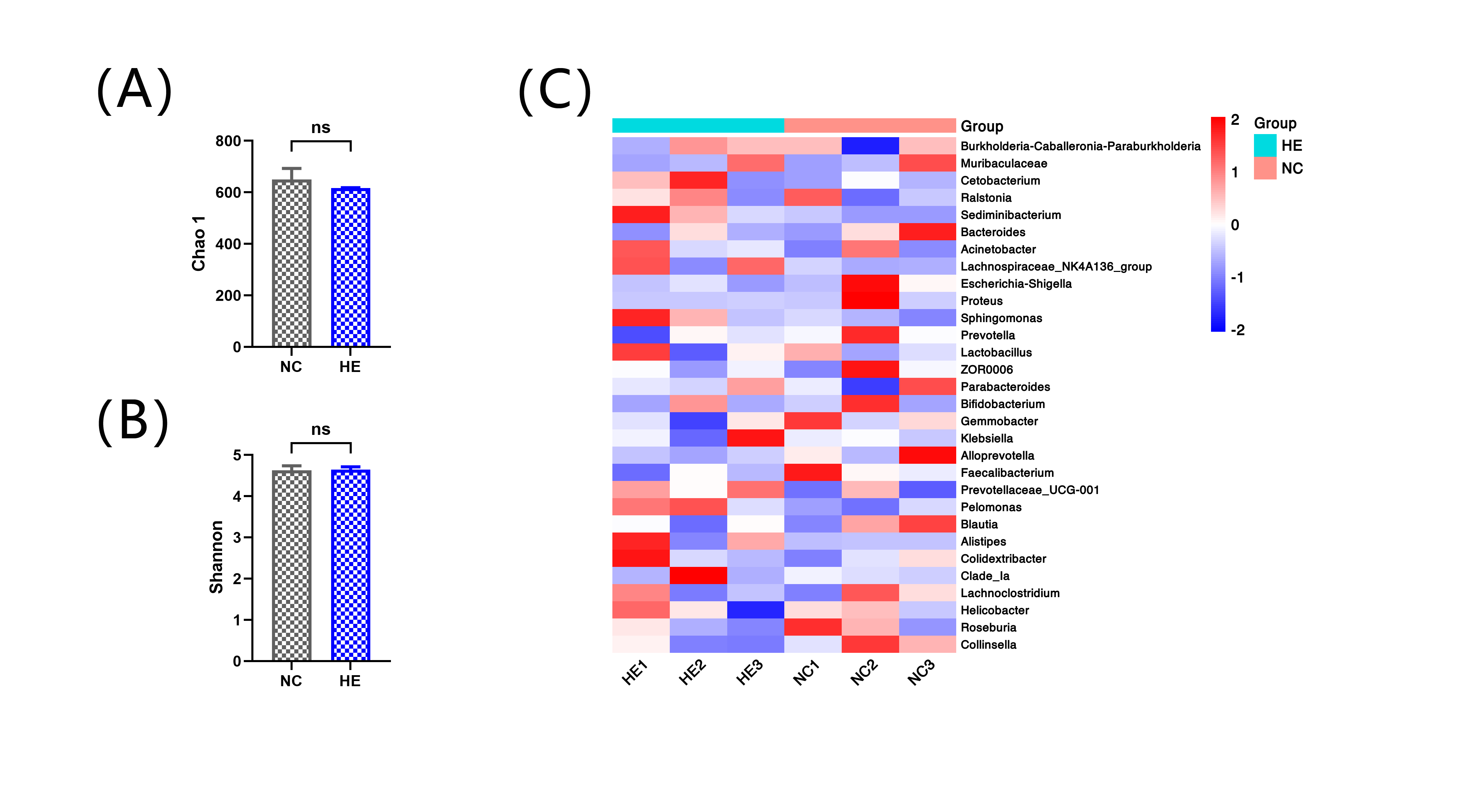

Supplement: Supplementary Figure S1 — Vaginal microbiota analysis in pregnant E18.5 HE and NC mice. (A,B) Chao 1 and Shannon indices (n = 3). (C) Heatmap of top 30 enriched genera. Error bars represent SEM. Significance determined by Student’s t-test. ns, not significant. [file Image_1.TIF]

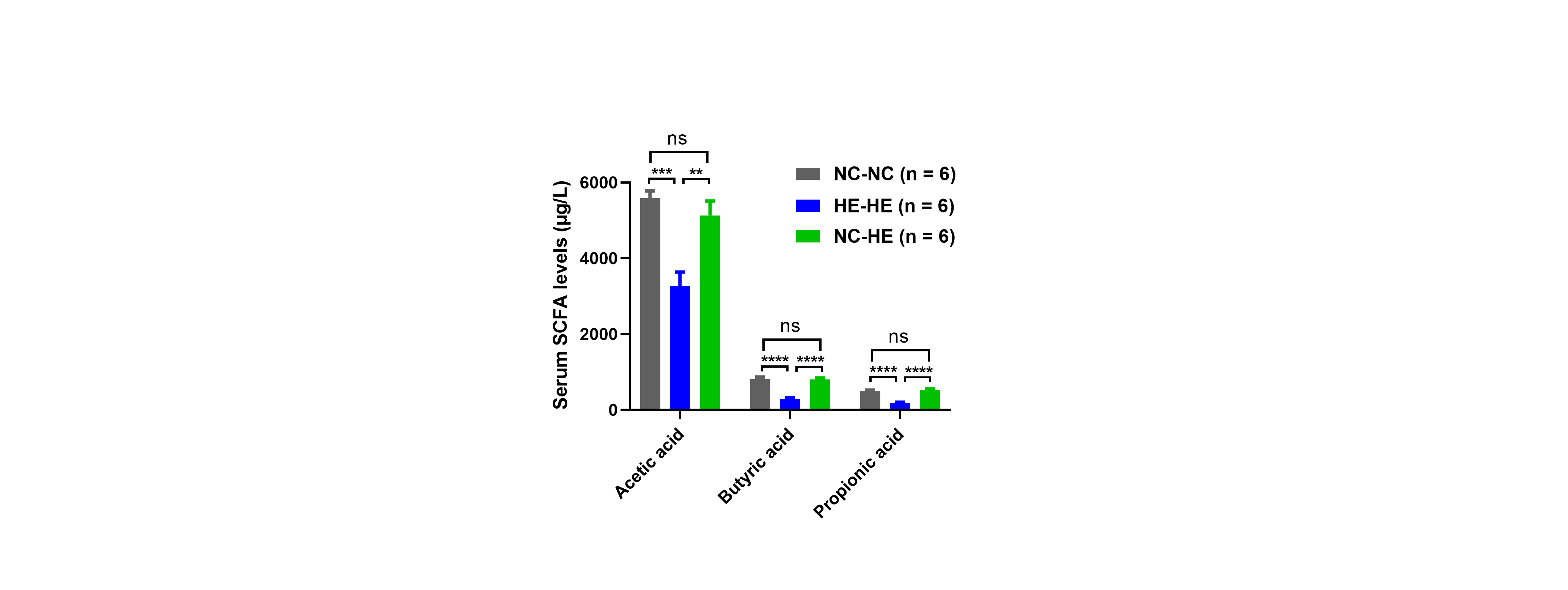

Supplement: Supplementary Figure S2 — Serum SCFA concentration in 24-week-old NC-NC, HE-HE, and NC-HE mice. Error bars represent SEM. Significance determined by one-way ANOVA. **p < 0.01; ***p < 0.001; ****p < 0.0001; ns, not significant. [file Image_2.TIF]
